# Segmentation of Rat Brains and Cerebral Hemispheres in Triphenyltetrazolium Chloride-Stained Images after Stroke †

**DOI:** 10.3390/s21217171

**Published:** 2021-10-28

**Authors:** Herng-Hua Chang, Shin-Joe Yeh, Ming-Chang Chiang, Sung-Tsang Hsieh

**Affiliations:** 1 Department of Engineering Science and Ocean Engineering, National Taiwan University, Taipei 10617, Taiwan; herbertchang@ntu.edu.tw; 2 Graduate Institute of Anatomy and Cell Biology, College of Medicine, National Taiwan University, Taipei 10051, Taiwan; shinjoeyeh@gmail.com; 3 Department of Neurology and Stroke Center, National Taiwan University Hospital, Taipei 10002, Taiwan; 4 Department of Biomedical Engineering, National Yang Ming Chiao Tung University, Taipei 11221, Taiwan; 5 Graduate Institute of Clinical Medicine, College of Medicine, National Taiwan University, Taipei 10051, Taiwan; 6 Graduate Institute of Brain and Mind Sciences, College of Medicine, National Taiwan University, Taipei 10051, Taiwan; 7 Center of Precision Medicine, College of Medicine, National Taiwan University, Taipei 10051, Taiwan

**Keywords:** image segmentation, brain extraction, hemisphere segmentation, superpixel, saliency map, parametric deformable model, gradient vector flow

## Abstract

Ischemic stroke is one of the leading causes of death among the aged population in the world. Experimental stroke models with rodents play a fundamental role in the investigation of the mechanism and impairment of cerebral ischemia. For its celerity and veracity, the 2,3,5-triphenyltetrazolium chloride (TTC) staining of rat brains has been extensively adopted to visualize the infarction, which is subsequently photographed for further processing. Two important tasks are to segment the brain regions and to compute the midline that separates the brain. This paper investigates automatic brain extraction and hemisphere segmentation algorithms in camera-based TTC-stained rat images. For rat brain extraction, a saliency region detection scheme on a superpixel image is exploited to extract the brain regions from the raw complicated image. Subsequently, the initial brain slices are refined using a parametric deformable model associated with color image transformation. For rat hemisphere segmentation, open curve evolution guided by the gradient vector flow in a medial subimage is developed to compute the midline. A wide variety of TTC-stained rat brain images captured by a smartphone were produced and utilized to evaluate the proposed segmentation frameworks. Experimental results on the segmentation of rat brains and cerebral hemispheres indicated that the developed schemes achieved high accuracy with average Dice scores of 92.33% and 97.15%, respectively. The established segmentation algorithms are believed to be potential and beneficial to facilitate experimental stroke study with TTC-stained rat brain images.

## 1. Introduction

A stroke is a medical condition in which the blood supply to part of the brain is interrupted or diminished and this prevents brain tissue from receiving oxygen and nutrients. Particularly, ischemic stroke, which accounts for the majority of strokes, is one of the leading causes of death among the aged population worldwide. Cerebral ischemia can induce many injuries including energy failure, intracellular calcium overload, and cell death, which eventually result in the loss of neurological functions and permanent disabilities [1]. To understand the mechanism of cerebral ischemia, evaluate the effect of therapeutic interventions, and study the scope of behavioral manifestations, experimental ischemic stroke models play an essential role. Among the various models, the middle cerebral artery occlusion (MCAO) model in rodents has been widely employed. In this model, the middle cerebral artery is occluded to cause brain tissue ischemia, which results in a massive amount of cell deaths, called the infarct.

**Figure 1:**
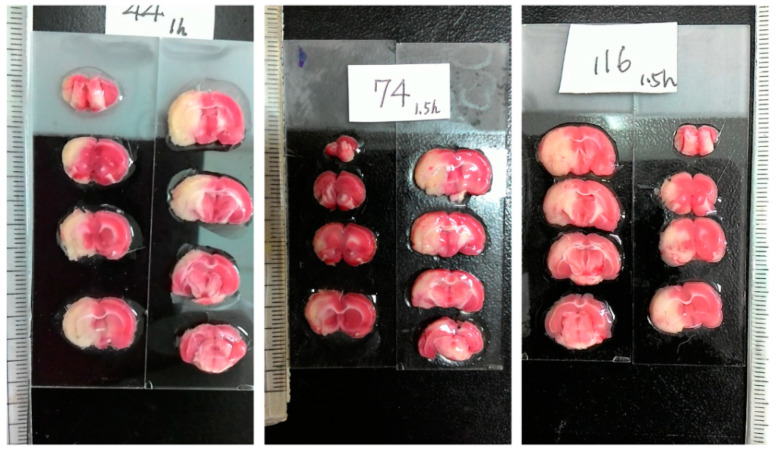
Representative examples of three camera-based TTC-stained rat brain images (900 × 1600) after ischemic stroke captured by a smartphone. Each image represents an individual rat subject with the MCAO model. Each image includes eight different rat brain slices, where the massive white regions indicate infarction.

In addition to magnetic resonance imaging (MRI) [2,3], 2,3,5-triphenyltetrazolium chloride (TTC) staining has been extensively employed to visualize the infarction for its celerity, veracity and cost effectiveness [4]. The colorless TTC reacts with the living cells, which results in the red compound, so that the red region essentially unveils the healthy/normal tissue. In contrast, the whiteness in the ischemic tissue reflects the absence of living cells, which generally indicates the infarct region. Consequently, TTC staining is powerful for the macroscopic differentiation between ischemic and non-ischemic tissue [5]. Requiring manual intervention, the image acquisition of TTC staining can be classified into two categories: scanner-based and camera-based. Figure 1 illustrates some typical camera-based TTC-stained rat brain images, which are captured using a modern smartphone. To process the TTC-stained rat brain images associated with the MCAO model, the first step is to extract the rat brain slices from the originally captured image, which usually contains a scale and a label. To further analyze the extracted brain slice, it is required to find the midline that separates the rat brain into two hemispheres.

Although there are abundant techniques in the literature for brain segmentation in medical images, the majority are dedicated to human brain investigation in MRI. Existing human brain segmentation methods with MR images can be broadly classified into six categories: mathematical morphology-based [6], image intensity-based [7], deformable model-based [8], anatomy atlas-based [9], deep learning-based [10] and hybrid methods [11]. Alternatively, some approaches are devoted to rodent brain extraction in MRI. For example, an automatic method based on the pulse coupled neural network was proposed to crop MR images of rat brain volumes [12]. An automatic brain extraction scheme that requires a brain template mask was introduced to extract the brain tissue in rat MR images [13]. Unfortunately, a specific computer-aided algorithm for TTC-stained rat brain image processing has been lacking. Manual delineation [4,14] and simple thresholding [15] of the rat brain and hemisphere regions on numerous images have been traditionally utilized. For example, Goldlust et al. [16] described a pipeline using thresholding and edge detection to segment the rat brain whereas a commercial image processing program (PhotoFinish, ZSoft Software) was adopted to manually draw the midline to bisect the brain. The Environment for Visualizing Images (ENVI 4.5) software associated with an image analysis tool (ImageJ, https://imagej.nih.gov/ij/) (accessed on 19 October 2021) were employed in [17] to process rat brain images. A semi-automated analysis program was proposed to segment rodent brain sections with TTC staining [18]. While manual delineation is time consuming, the outcome by thresholding approaches is inaccurate and case-dependent, which requires heavier human intervention to complete the segmentation task. To lessen the laborious burden and obtain accurate brain segmentation, an automatic and reliable method for brain extraction and hemisphere segmentation in TTC-stained rat brain images is fundamental.

We have conducted a preliminary study of brain extraction and hemisphere segmentation in TTC-stained rat brain images based on simple saliency detection associated with edge detection and morphological operations [19]. From a different perspective, this paper attempts to develop fully automatic algorithms to address the problems of brain extraction and hemisphere segmentation starting from initially captured TTC-stained rat brain images as shown in Figure 1. The proposed framework consists of two consecutive stages that correspond to the two different tasks. In the first stage of brain extraction, a superpixel oversegmentation associated with salient region detection algorithm is proposed to efficiently segment the brain slices, followed by a parametric deformable model for accuracy improvement. In the second stage of hemisphere segmentation, the midline of the brain is obtained through brain edge detection and initial midline estimation followed by an advanced deformable model for midline refinement. In summary, there are five and three phases for the brain extraction and hemisphere segmentation tasks, respectively. In contrast to our prior investigation [19], considerably distinct approaches are developed except for the first phase of oversegmentation in the brain extraction mission. The major contributions of the current work are summarized as follows:

1. Challenges to brain extraction and hemisphere segmentation in TTC-stained rat images captured by a smartphone are discussed.
2. An automatic rat brain extraction algorithm in light of saliency region detection and active contour rectification is investigated.
3. An automatic rat hemisphere segmentation scheme based on initial midline estimation refined by the gradient vector flow is introduced.
4. Influences of light reflection and brain distortion on the segmentation accuracy are reduced due to the proposed frameworks.
5. Massive experiments in fair comparison with competitive methods are administered for segmentation performance evaluation.
6. A computer-aided tool is provided for closer monitoring of the rat brain region.
7. Overall rat brain processing time is reduced in contrast to manual delineation.

## 2. Brain Extraction

### 2.1. Challenges

As illustrated in Figure 1, the extraction of the individual rat brain slices from the originally captured TTC-stained image by a smartphone is challenging according to the following observations:

1. In addition to the stained rat brain slices, there are a scale and a label indicating the status of the subject being experimented, both of which need to be eliminated.
2. The shape of the brains is irregular with broken and ambiguous boundaries.
3. The colors of the brain slices range from white, pink, to cardinal with a nonuniform distribution.
4. There are some bright stains on the brain regions due to the reflection of the moisture in the organ.
5. The background is not clean and simple with a varying intensity distribution and a complicated pattern of light reflection.

To address these challenges, an efficient rat brain extraction algorithm in TTC-stained images is proposed, which consists of superpixel oversegmentation, salient feature computation, saliency trimap construction, salient region extraction, and final brain segmentation, as described in the following.

### 2.2. Superpixel Oversegmentation

A superpixel is a perceptually meaningful region that comprises several pixels with prescribed conditions, which can be employed to replace the strict structure of the pixel grid. Features acquired from superpixels have been shown to be effective and efficient for salient object detection [20,21]. Salient region detection has been successfully applied to many image-processing applications including segmentation [22]. As such, our approach first segments the TTC-stained rat brain image so that the segmented elements exhibit similar color characteristics with comparable dimensions and salient boundaries. To achieve this, we perform oversegmentation on the TTC-stained image to establish the set of superpixels $Z=\left\{z_{1},\cdots ,z_{K}\right\}$ , where $z_{i}$ represents the i th superpixel and K is the total number of superpixels being constructed. Considering its high efficiency and low complexity, we utilize the simple linear iterative clustering (SLIC) method [23], which employs a *k*-means clustering technique to produce superpixels. The number of superpixels is empirically set as K=65 to facilitate the subsequent process.

### 2.3. Salient Feature Computation

To retrieve each individual brain slice from the initial oversegmentation map, we compute a series of salient features for each superpixel $z_{i}$ . Since our biological vision system is highly perceptive to color contrast, a histogram-based contrast scheme is introduced to define saliency values based on color statistics. Let $c_{i}$ denote the color of the i th superpixel $z_{i}$ ; the global color saliency is defined in light of its color contrast to all other superpixels as

(1) $$GCS\left(z_{i}\right)=\overset{K}{\underset{j=1,i\neq j}{\sum }}\Delta \left(c_{i},c_{j}\right)$$

where $GCS\left(z_{i}\right)$ represents the global color saliency of $z_{i}$ and $\Delta \left(c_{i},c_{j}\right)$ indicates the color distance metric between $c_{i}$ and $c_{j}$ that corresponds to superpixels $z_{i}$ and $z_{j}$ respectively. The metric $\Delta \left(c_{i},c_{j}\right)$ is computed according to the Euclidean distance between the i th and the j th superpixels in both the RGB and CIELab color spaces. Specifically, there are six dimensions in the color distance computation to accommodate sharp color variation in the TTC-stained brain image.

In addition to the global color saliency, a local color saliency feature is defined as

(2) $$LCS\left(z_{i}\right)=\overset{K}{\underset{j=1,i\neq j}{\sum }}\alpha \left(p_{i},p_{j}\right)\Delta \left(c_{i},c_{j}\right)$$

where $LCS\left(z_{i}\right)$ represents the local color saliency of superpixel $z_{i}$ and $\alpha \left(p_{i},p_{j}\right)$ denotes the local proximity weight with

(3) $$\alpha \left(p_{i},p_{j}\right)=\frac{1}{N_{p}}\exp\left(-\frac{1}{2\sigma_{p}^{2}}\|p_{i}-p_{j}{\|}^{2}\right)$$

where $N_{p}$ is a normalization term, $\sigma_{p}$ is the standard deviation, and $p_{i}$ and $p_{j}$ are the coordinates of superpixels $z_{i}$ and $z_{j}$ respectively. For long distance pairs, the value of $\alpha \left(p_{i},p_{j}\right)$ is small, the local color saliency feature would be similar to the central peripheral contrast. If $\alpha \left(p_{i},p_{j}\right)$ is constant, $LCS\left(z_{i}\right)$ is analogous to the local contrast in [24]. In contrast to the global color saliency, the local color saliency introduces the proximity weight to reinforce the relevance of neighboring superpixels. This is advantageous to aggregate adjacent superpixels into a large component in the brain region while reducing the light reflection effect.

As the histogram is one of the most powerful saliency measures, we integrate color histogram characteristics into a histogram-based contrast feature. This color histogram feature is defined using the chi-square distance between different histogram pairs using quantized colors, i.e.,

(4) $$CHC\left(z_{i}\right)=\overset{K}{\underset{j=1}{\sum }}\overset{B}{\underset{k=1}{\sum }}\frac{{\left(h_{i}\left(k\right)-h_{j}\left(k\right)\right)}^{2}}{h_{i}\left(k\right)+h_{j}\left(k\right)}$$

where $CHC\left(z_{i}\right)$ represents the color histogram contrast of superpixel $z_{i}$ , B is the total number of histogram bins, and $h_{i}\left(k\right)$ and $h_{j}\left(k\right)$ indicate the k th histogram bins of superpixels $z_{i}$ and $z_{j}$ respectively.

Based on the observation of the TTC-stained rat brain images, the color of the background is generally more diversified whereas the foreground color is more concentrated, which implies saliency. To understand the occurrence probability of each superpixel, a feature pertinent to the spatial coordinate distribution of superpixels is defined as

(5) $$SCD\left(z_{i}\right)=\overset{K}{\underset{j=1}{\sum }}\beta \left(c_{i},c_{j}\right)\|p_{j}-\mu_{i}{\|}^{2}$$

where $SCD\left(z_{i}\right)$ represents the spatial coordinate distribution of superpixel $z_{i}$ and $\mu_{i}$ symbolizes the weighted mean position of $z_{i}$ , which is computed using

(6) $$\mu_{i}=\overset{K}{\underset{j=1}{\sum }}\alpha \left(p_{i},p_{j}\right)$$

and $\beta \left(c_{i},c_{j}\right)$ indicates the color affinity weight between the colors of superpixels $z_{i}$ and $z_{j}$ , which is similar to the form of $\alpha \left(p_{i},p_{j}\right)$ with

(7) $$\beta \left(c_{i},c_{j}\right)=\frac{1}{N_{c}}\exp\left(-\frac{1}{2\sigma_{c}^{2}}\|c_{i}-c_{j}{\|}^{2}\right)$$

where $N_{c}$ is a normalization term and $\sigma_{c}$ is the standard deviation for controlling the shape of the function.

### 2.4. Saliency Trimap Construction

Once the saliency features for all superpixels are computed, a classification algorithm is employed to inspect whether each region is salient or not. In respect to effectiveness and generalization, the random forest classifier [25] is exploited. A random forest or random decision forest is an ensemble learning scheme mainly for classification and regression. By constructing multiple decision trees at training time, it operates in such a way that each individual tree generates a class indicator and the class with the most votes grows into the model’s prediction. The fundamental concept behind the random forest is to integrate the bootstrap aggregating idea with random feature selection to maximize the prediction accuracy. Specifically, the code provided in [26] is adopted for our random forest classification implementation, which is trained using the image dataset with labeled images as described in [27]. A three-class classification design is utilized because the trimap strategy has been commonly manipulated in matting [28] and saliency [29] methods. In our approach, a superpixel is judged by two different thresholds to generate the trimap. If the prediction value of a superpixel is larger than $T_{f}$ , where $T_{f}$ denotes the threshold for the foreground, it is designated to the foreground class. Alternatively, if the prediction outcome is smaller than $T_{b}$ , where $T_{b}$ signifies the threshold for the background, the superpixel belongs to the background class. However, if the prediction score is in between $T_{f}$ and $T_{b}$ , it is regarded as the unidentified candidate. Comparing to the strict binary map, the trimap with an additional ambiguous class provides more reliable and flexible segmentation of salient regions in the TTC-stained rat brain images.

### 2.5. Salient Region Extraction

The construction of the trimap is for initial saliency estimation, which requires further processing to refine the three-class classification into rigid foreground and background segmentation. This is achieved by the use of the *SaliencyCut* algorithm [30], which takes the computed saliency trimap to facilitate automatic salient region segmentation. Founded on the GrabCut [31], the SaliencyCut accomplishes automation by two enhancements: iterative refining and adaptive fitting. Particularly, the SaliencyCut algorithm iteratively refines the initial salient regions to handle noisy initialization given a high recall of potential foreground regions and enables the iterative optimization process to boost the precision. Based on the observation that regions closer to an initial salient object are presumably part of that object, the SaliencyCut adaptively adjusts the initial condition to fit with newly segmented salient regions. As such, the new initialization scheme in the SaliencyCut allows the GrabCut to include adjacent salient superpixels and exclude non-salient superpixels in the trimap of the TTC-stained rat brain image. After each GrabCut iteration, the algorithm incorporates the restraints provided by the updated trimap into consideration to output the final segmentation.

### 2.6. Final Brain Segmentation

After obtaining the TTC-stained image segmentation outcome, the rat brain regions indicating saliency are often with ragged boundaries. This is primarily due to the fact of inevitable bright stains inside the brain regions and around the brain surfaces, which are caused by the light reflection effect. These bright stains exhibit quite a different color tone from the brain so that they are excluded from the salient regions, i.e., the segmented brain slices. In consequence, the interior bright stains result in holes in the brain regions, which are resolved with a morphological hole-filling process

(8) $$X_{k}=\left(X_{k-1}⊕E_{h}\right)\cap M_{s}^{c}\;\;\;k=1,\;2,\;3,\ldots$$

where ⊕ symbolizes the dilation operator, $E_{h}$ is a 3×3 symmetric structuring element of cross, and $M_{s}^{c}$ is the complement of $M_{s}$ , which is the binary mask produced from the salient region extraction process. This procedure terminates at iteration step k when $X_{k}=X_{k-1}$ , where $X_{k}$ contains all the filled holes in $M_{s}$ . The final segmentation mask without holes is attained by superimposing $X_{k}$ on $M_{s}$ :

(9) $$M_{h}=X_{k}\cup M_{s}$$

where ∪ denotes the union operator and $M_{h}$ is the improved segmentation mask without interior holes.

To tackle the ragged boundary problem, a parametric deformable model is suggested. Thanks to its simplicity and popularity, the parametric active contour (also known as Snakes) [32], which has been broadly applied in image segmentation and object tracking, is exploited. A snake is defined as a set of ordered points or snaxels v(s)=[x(s), y(s)] , s∈[0, 1] , which are usually generated counter-clockwise. A parametric active contour advances through the spatial domain of an image to minimize the energy functional

(10) $$E_{snake}=\int_{0}^{1}E_{int}\left(v\left(s\right)\right)+E_{ext}\left(v\left(s\right)\right)ds$$

where $E_{int}$ and $E_{ext}$ denote the internal energy and external energy, respectively. The internal energy in Equation (10) is defined as

(11) $$E_{int}\left(v\left(s\right)\right)=\alpha \left(s\right){\left|v_{s}\right|}^{2}+\beta \left(s\right){\left|v_{ss}\right|}^{2}$$

where α(s) and β(s) are weighting functions that control the tension and rigidity of the contour, respectively. The first order derivative $v_{s}$ with respect to s adjusts the distance between adjacent snaxels. During energy minimization, the second order term $v_{ss}$ enables the contour to resist flection.

Alternatively, the external energy in Equation (10) is designed to let the contour interact with the image. To handle the blurred boundaries of the rat brain in the TTC-stained image, the segmented color image $I_{c}$ after the hole filling process is transformed using

(12) $$\hat{I}\left(x,y\right)=\{\begin{array}{cc} 2I_{r}\left(x,y\right)-I_{g}\left(x,y\right)-I_{b}\left(x,y\right) & \mathrm{if}\;\left|I_{r}\left(x,y\right)-I_{b}\left(x,y\right)\right|\leq T_{s}\\ 2I_{r}\left(x,y\right)-I_{g}\left(x,y\right)+I_{b}\left(x,y\right) & \mathrm{otherwise} \end{array}$$

where $\hat{I}$ is the transformed gray scale image; $T_{s}$ is a threshold; and $I_{r}$ , $I_{g}$ and $I_{b}$ are the red, green and blue channels of $I_{c}$ respectively. Since the rat brain is primarily red, for the blurred brain boundaries the absolute difference between the red and blue channels should be relatively small. As such, the intensity in the brain regions is boosted whereas the intensity in the blurred boundaries is diminished. Subsequently, the external energy is derived from $\hat{I}$ using

(13) $$E_{ext}\left(x,y\right)=-\gamma {\left|\nabla \left[G_{\sigma }\left(x,y\right)*\hat{I}\left(x,y\right)\right]\right|}^{2}$$

where γ is the weight, ∇ symbolizes the gradient operator, ∗ denotes the convolution operation and $G_{\sigma }\left(x,y\right)$ represents a 2-D Gaussian function with a standard deviation σ . The boundary of the segmented brain mask after hole filling is utilized for the initial contour of the proposed deformable model. The contour evolves towards the brain surface based on Equation (10) until convergence is achieved, where the entire rat brain extraction procedure is completed. The extracted brain slices are saved in each individual image with a fixed dimension to facilitate subsequent processing.

## 3. Hemisphere Segmentation

### 3.1. Challenges

After extracting the rat brain slices, the next stage is performing hemisphere segmentation, which is also challenging as detailed in the following:

1. Due to the manual placement of the rat brain slices, the midline is randomly oriented, not vertically.
2. The rat brain can be seriously distorted due to the infarction of the induced stroke so that the midline is convoluted.
3. The midline exhibits a similar color tone to its surrounding tissues and is visible in short segments to the naked eyes.
4. There are merely few anatomically salient structures around the midline that can provide meaningful information for the identification.

We tackle the rat hemisphere segmentation problems with the described difficulties using a series of image processing steps containing medial subimage extraction, initial midline detection, and final midline estimation.

### 3.2. Medial Subimage Extraction

**Figure 2:**
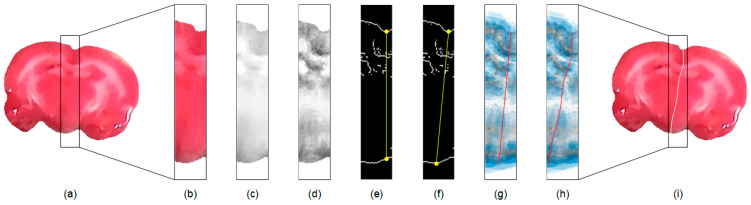
Illustration of the proposed rat hemisphere segmentation algorithm. (**a**) Extracted rat brain slice. (**b**) Segmented medial subimage from (**a**). (**c**) Red channel image $S_{r}$ of (**b**). (**d**) Enhanced image $S_{c}$ of (**c**). (**e**) Edge map $S_{e}$ of (**d**). (**f**) Initial straight midline connecting the brain boundaries. (**g**) Initial midline superimposed on the GVF field. (**h**) Estimated midline after curve evolution. (**i**) Final midline superimposed on the input image for separation.

Since the midline is across the medial part of the brain, an efficient approach for hemisphere segmentation is to restrict the processing region to the proximity of the midline. To achieve this, in each extracted rat brain image, e.g., Figure 2a, we first compute the center of mass using the corresponding binary mask. A rectangular subimage containing the medial brain based on the coordinates of the center of mass with a width, w is then computed as illustrated in Figure 2b. Herein, an appropriate size is empirically determined by setting w=36 pixels. In our experience, this setting suffices for the inclusion of most midlines in the extracted subimage. If necessary, the value of w can be increased to involve greatly convoluted midlines. To facilitate the subsequent processing, in Figure 2c, only the red channel of the TTC-stained subimage is utilized, which is denoted as $S_{r}$ . This gray scale image is further enhanced using the adaptive histogram equalization method [33] to increase the contrast as shown in Figure 2d and denoted as $S_{c}$ .

### 3.3. Initial Midline Detection

Subsequently, the edge map of $S_{c}$ is computed using the Sobel operator and denoted as $S_{e}$ . The edge map presents apparent brain borders, which are managed for the initial midline estimation. The computation starts from the searching of the superior groove and inferior concave points in the edge map of the subimage as illustrated in Figure 2e. In our approach, the superior groove point is detected by searching for the lowest position from the highest location along the superior brain boundary towards the medial axis. A vertical line is drawn from the identified groove point to intersect the inferior brain boundary. The inferior concave point is then detected by exploring the deepest position with the longest length around the intersection to accommodate distorted brain shapes. As depicted in Figure 2f, an initial straight midline is generated from the superior groove point to the inferior concave point for further evolution.

### 3.4. Final Midline Estimation

Because the cerebral hemispheres are rarely symmetric, especially in ischemic rat brains, the preceding midline usually provides no accurate estimation of the midsurface that separates the rat brain. A parametric deformable model that is derived from snakes [32], which is called the gradient vector flow (GVF) [34], is exploited to address this issue. The GVF improves the traditional snake model by introducing the GVF field, which is a vector field with v(x,y)=[u(x,y),v(x,y)] that minimizes the energy functional

(14) $$E_{GVF}=\iint^{\;}\mu_{g}\left(u_{x}^{2}+u_{y}^{2}+v_{x}^{2}+v_{y}^{2}\right)+{\left|\nabla S_{g}\right|}^{2}{\left|v-\nabla S_{g}\right|}^{2}dxdy$$

where $\mu_{g}$ is a regularization parameter controlling the weight between the first term and the second term in the integrand; $S_{g}\left(x,y\right)$ is an edge map produced from the input subimage $S_{c}\left(x,y\right)$ ; and u(x,y) and v(x,y) are the vector field component in the x - and y - axes, respectively. An open GVF contour is initialized using the straight midline as delineated in Figure 2g. To guide the contour advancing towards the rat brain midsurface, the GVF field is computed by solving the following Euler equations

(15) $$\mu_{g}\nabla^{2}u-\left(u-{\left(S_{g}\right)}_{x}\right)\left({\left(S_{g}\right)}_{x}^{2}+{\left(S_{g}\right)}_{y}^{2}\right)=0\;\mu_{g}\nabla^{2}v-\left(v-{\left(S_{g}\right)}_{y}\right)\left({\left(S_{g}\right)}_{x}^{2}+{\left(S_{g}\right)}_{y}^{2}\right)=0$$

where $\nabla^{2}$ indicates the Laplacian operator. The GVF field is interpolated from the image edge map that reflects a kind of competition among the boundary vectors, which helps restrict the curve evolution inside $S_{c}$ . When the stopping criterion is reached, the contour locates in the proximity of the midsurface as shown in Figure 2h. A morphological operation of thinning is applied to the GVF curve to produce a one-pixel-wide contour to reinforce segmentation accuracy, which is superimposed on the input image to separate the rat brain as illustrated in Figure 2i. The abovementioned procedures are independently applied to each extracted rat brain TTC-stained image.

## 4. Results and Discussion

### 4.1. Implementation and Image Acquisition

The proposed brain extraction and hemisphere segmentation algorithms in rat TTC-stained images were implemented and programmed in MATLAB R2019a (The MathWorks Inc. Natick, MA, USA). All experiments were executed on an Intel^®^ Core (TM) i5-3210M CPU @ 2.50GHz with 8 GB RAM running 64-bit Windows 10. The deformable model parameters were set as follows: α=1.4 , β=1.4 , γ=1 and $\mu_{g}=0.2$ . This study employed an ischemia-reperfusion model based on MCAO on the right side with a silicon-coated nylon filament. Different ischemic durations were designed to develop a wide range of infarcts. Male *Sprague-Dawley* rats with ages of 7–9 weeks old and body weights of 180–340 g were adopted as experimental specimens. There were 40 rats sacrificed for in vitro TTC staining to evaluate the proposed brain extraction and hemisphere segmentation algorithms. Each stained rat brain was cut into eight slices along the coronal direction with each slice 2 mm in thickness. The experiments were carried out in accordance with the principles of the Basel Declaration. All eight stained slices were coded and photographed together using a modern smartphone (Zenfone Z00D, ASUSTeK Computer Inc., Taipei, Taiwan) located at a fixed position for the experiments as illustrated in Figure 1.

### 4.2. Evaluation Metrics

We computed different similarity metrics between the segmentation and gold standard masks to evaluate the performance of the proposed brain extraction and hemisphere segmentation frameworks. The gold standard outlines of the brains and hemispheres were delineated by an experienced neurologist in our team while referring to the rat brain atlas. Particularly, the conformity metric $\kappa_{c}$ was adopted to evaluate the overall accuracy by measuring the unity subtracted by the ratio of the number of mis-segmented pixels to the number of correctly segmented pixels using [35]

(16) $$\kappa_{c}=1-\frac{\theta_{FP}+\theta_{FN}}{\theta_{TP}}\times 100\%$$

where $\theta_{FP}$ indicates false positives, $\theta_{FN}$ indicates false negatives, and $\theta_{TP}$ indicates true positives. Two other global similarity metrics, Jaccard $\kappa_{J}$ [36] and Dice $\kappa_{D}$ [37], were also utilized to understand the segmentation performance with

(17) $$\kappa_{J}=\frac{\theta_{TP}}{\theta_{TP}+\theta_{FN}+\theta_{FP}}\times 100\%$$

and

(18) $$\kappa_{D}=\frac{2\theta_{TP}}{2\theta_{TP}+\theta_{FN}+\theta_{FP}}\times 100\%$$

Additionally, two local similarity metrics of sensitivity $\eta_{st}$ and sensibility $\eta_{sb}$ [35] were employed to evaluate the degree of under-segmentation and over-segmentation using

(19) $$\eta_{st}=\frac{\theta_{TP}}{\theta_{TP}+\theta_{FN}}\times 100\%$$

and

(20) $$\eta_{sb}=1-\frac{\theta_{FP}}{\theta_{TP}+\theta_{FN}}\times 100\%$$

Finally, a pixel number difference measure $\Delta_{N}$ was included to compute the absolute difference between the pixel number of the segmentation result ${Ν}_{s}$ and the pixel number of the gold standard ${Ν}_{g}$ with

(21) $$\Delta_{N}=\left|{Ν}_{s}-{Ν}_{g}\right|$$

Another pixel number distinction metric $\Delta_{F}$ was adopted to realize the number of wrong pixels with spatial correlation:

(22) $$\Delta_{F}=\theta_{FP}+\theta_{FN}$$

A pixel number error ratio metric $\varepsilon_{F}$ indicating the percentage of the pixel number distinction to the ground truth pixel number was exploited to understand the overall segmentation error with

(23) $$\varepsilon_{F}=\frac{\Delta_{F}}{{Ν}_{g}}\times 100\%$$

### 4.3. Evaluation of Rat Brain Extraction

**Figure 3:**
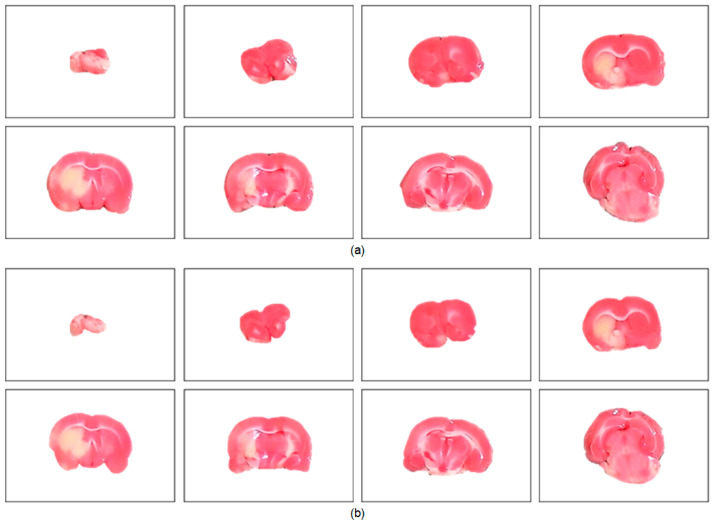
Visual evaluation of the rat brain extraction results of Subject 37. There are eight brain slices extracted from a large compound image (Figure 1) and stored in the same 480 × 320 image. (**a**) The proposed algorithm. (**b**) The gold standard.

**Figure 4:**
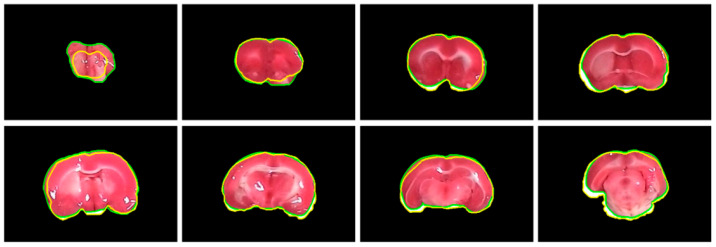
Visual comparison of subject 20 between our rat brain extraction contours (green) and the gold standard outlines (yellow). The brain extraction results were superimposed on the gold standard images with a dimension of 480 × 320 for all slices.

The performance of the proposed brain extraction algorithm in the experimental image data was first evaluated. The input images were the original camera-based TTC-stained rat brain slices as illustrated in Figure 1. The segmentation goal is to generate eight individual brain slices per specimen from the big photographic images with complicated scenes. Figure 3 visually demonstrates the rat brain extraction results of a representative example (Subject 37) in comparison with the gold standard slices. To facilitate subsequent processing, each brain slice was stored in a 480 × 320 image. It was noted that the extracted brain slices in Figure 3a overcame the influences of light reflection and exhibited quite clean boundaries and complete structures. Another brain extraction instance (Subject 20) is depicted in Figure 4, where the green contours represent the segmented brain boundaries and the yellow contours indicate the gold standard outlines. The two different contours mostly overlapped each other but with apparent distinction in some segments. The separation distances between our automatic extraction curves and the gold standard outlines were relatively larger as the brain regions were getting smaller, especially for the first two slices. This is mainly because the rat brain is roughly an ellipsoid with varied cross sections. The inevitable thickness inherited in each cut brain slice results in two different brain sections being captured, where the top brain region is the desired target not the bottom brain section. Particularly for the first slice, which belongs to the olfactory bulb, the upper brain section is considerably smaller than the bottom brain section due to the rapidly changed geometry. Since the distinction between the bottom brain section and the background is stronger than the discrimination between the top and bottom brain sections, the proposed rat brain extraction algorithm mistakenly selected the bottom brain boundary as the segmentation output.

**Figure 5:**
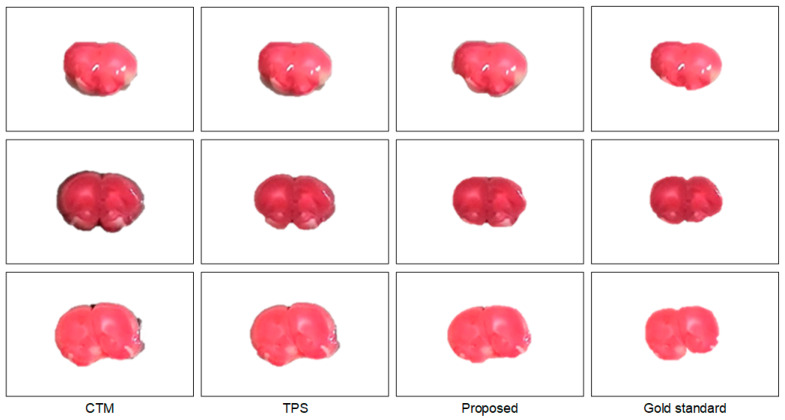
Visual comparison of brain extraction results in rat TTC-stained images using different methods. Top row: slice 1 of Subject 2. Middle row: slice 2 of Subject 20. Bottom row: slice 3 of Subject 37.

To further understand the characteristics of the proposed algorithm and demonstrate the difficulty of rat brain extraction in TTC-stained images, we compared our segmentation results with two different approaches: one is the color thresholding method (CTM) [38] and the other is the two phase segmentation (TPS) using active contours [39]. Without available codes of rat brain extraction methods for comparison, the reasons for choosing these two methods are that the simple CTM provides a benchmark whereas the popular TPS enables perceiving the improvement with an advanced scheme. Figure 5 illustrates the extracted brain slices of three different subjects using the CTM, TPS and proposed methods along with the gold standard. The segmentation results produced by the CTM included visible background pixels around the brain surfaces, which led to relatively larger brain areas. Fewer background pixels were observed in the TPS brain slices comparing to the CTM results. It was our rat brain extraction algorithm that outperformed the competitive methods generating the brain slices that best resembled the gold standard in all scenarios.

**Figure 6:**
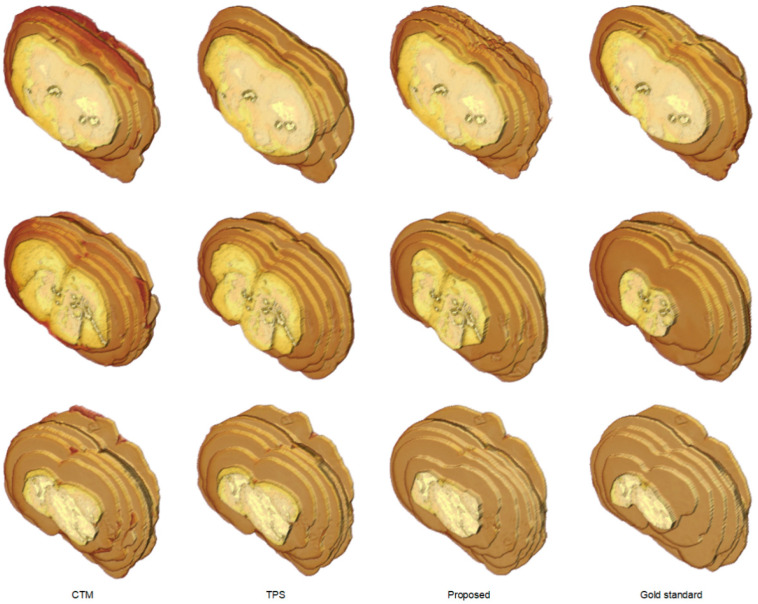
Visual comparison of rat brain extraction results in TTC-stained images using different methods in 3-D view. Top row: Subject 2. Middle row: Subject 20. Bottom row: Subject 37.

The segmentation outcomes of all brain slices of the representative examples in Figure 5 were demonstrated in Figure 6 with 3-D view. While the CTM results exhibited larger geometry differences to the gold standard, the difference was diminished by the TPS method and further reduced by the proposed framework. Table 1 presents the segmentation accuracy in terms of $\kappa_{D}$ for the brain extraction outcomes in Figure 5 and Figure 6, where our scheme obtained the highest scores in all instances. Quantitative performance assessment of all experimented methods using the 40 rat brain subjects in the acquired dataset in light of five different evaluation metrics was summarized in Table 2. Both CTM and TPS methods produced high average $\eta_{st}$ scores but low average $\eta_{sb}$ values, which corresponded to the observation in Figure 5 and Figure 6 that excessive background regions were involved in the segmentation results. The proposed rat brain extraction algorithm, in contrast, provided a much higher average $\eta_{sb}$ score than the CTM and TPS methods, which resulted in the greatest average $\kappa_{c}$ value. Indeed, our segmentation scheme yielded the best average scores in all performance evaluation metrics. Nevertheless, the efficacy of the proposed method can be deteriorated if the light reflection region is tightly connected to the brain boundaries or the light reflection region inside the brain produces a deep cave. The computation times were approximately 0.53 s, 1.67 s and 1.52 s for the CTM, TPS and proposed methods, respectively.

### 4.4. Evaluation of Rat Hemisphere Segmentation

**Figure 7:**
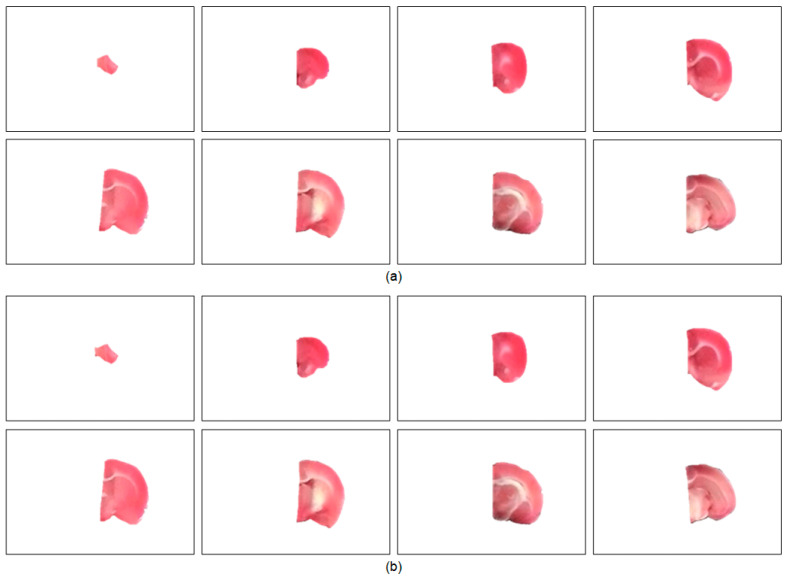
Visual evaluation of the left hemisphere segmentation results of Subject 16. (**a**) The proposed algorithm. (**b**) The gold standard. The $\kappa_{D}$ values were 93.06%, 98.29%, 97.92%, 98.22%, 98.51%, 97.53%, 99.02% and 96.52% from top left to bottom right, respectively.

**Figure 8:**
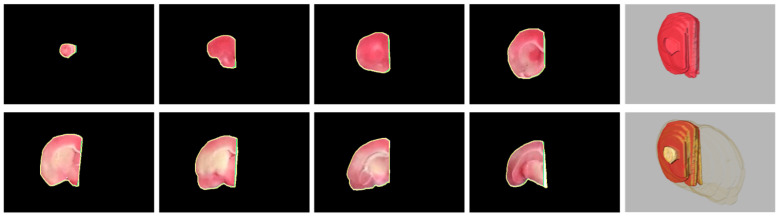
Visual comparison between the rat hemisphere segmentation contours (green) and the gold standard outlines (yellow). From top left, the $\kappa_{c}$ values were 88.58%, 97.58%, 97.01%, 97.24%, 98.03%, 96.26%, 98.78% and 92.79%, respectively. Top right: 3-D view of the segmented right hemisphere, $\kappa_{c}=96.77\%$ . Bottom right: 3-D view of the segmented hemisphere superimposed with the gold standard, $\kappa_{D}=98.41\%$ .

After accomplishing the individual rat brain slices, an important task is to separate the brain into two hemispheres for further processing and analysis. Figure 7 illustrates the split left brain regions of a representative specimen using the proposed hemisphere segmentation algorithm along with the gold standard hemisphere. The segmented left brain slices approximately conformed to the gold standard brain surfaces with a volumetric $\kappa_{D}=97.91\%$ . Figure 8 shows the eight separated right brain regions and their 3-D view of the same subject in Figure 7, where the segmentation contours overlapped the gold standard contours. The midlines estimated by our framework were quite close to the midlines delineated by the expert in most brain slices, leading to high evaluation scores of $\eta_{st}=97.03\%$ , $\eta_{sb}=99.83\%$ and $\kappa_{c}=96.77\%$ . It was noted that some midlines were convoluted due to the distorted brain caused by the infarction, which increased the difficulty of accurate segmentation. Since rat hemisphere segmentation in TTC-stained images is a particular task and, to the best of our knowledge, no other available code has been publically released for comparison, the uniquely developed scheme for our dataset was evaluated by computing the abovementioned performance measure metrics based on the gold standard. Table 3 presents the quantitative similarity evaluation scores of our rat hemisphere segmentation results in the TTC-stained image dataset. The segmentation accuracy of both left and right hemispheres was comparable in all performance measure metrics, which exhibited high overall average values of $\kappa_{D}=97.15\%$ , $\eta_{st}=96.51\%$ and $\eta_{sb}=97.83\%$ . Finally, Table 4 summarized our hemisphere segmentation error analyses, where the left hemisphere outcome indicated a lower average $\Delta_{N}$ score whereas the right hemisphere outcome revealed a smaller average $\Delta_{F}$ score. The overall volumetric segmentation error attained a low average $\varepsilon_{F}=5.66\%$ .

Thanks to the stimulation of the assistance in experimental stroke investigation with TTC-stained rat images, we exclusively developed brain extraction and hemisphere segmentation algorithms in this work. To manage the raw TTC-stained image data captured by a smartphone and to handle the light reflection and background noise issues caused by the camera sensor, the brain extraction framework consisted of five different processing phases. By aggregating similar pixels into superpixels, the subsequent application of the salient region extraction scheme efficiently separated the brain foreground from the complicated background while reducing the influence of the sensor-related headaches. To eliminate the thickness regions contained in the initial brain extraction results, the parametric deformable model associated with the color image transformation scheme adequately achieved rat brain refinements as demonstrated in Figure 3 and Figure 4. Qualitative and quantitative comparisons between the proposed approach and other competitive methods further validated the effectiveness of our tactics as presented in Figure 5 and Figure 6 as well as Table 1 and Table 2, respectively. On the other hand, three different phases were designed in the hemisphere segmentation framework to deal with the difficulty of the midline computation. By restricting the estimation in the medial subimage followed by the GVF model, more accurate hemisphere segmentation results were acquired as shown in Figure 7 and Figure 8, and evaluated in Table 3 and Table 4. Lastly, the average hemisphere segmentation processing time was 0.85 s.

## 5. Conclusions

Devoted to brain extraction and hemisphere segmentation in TTC-stained rat images, this paper introduced various strategies to tackle these two individual tasks. To diminish the influence caused by the optical and sensor-related issues, the proposed rat brain extraction algorithm was founded on the integration of superpixel saliency detection and parametric contour segmentation. For rat hemisphere segmentation, open curve evolution guided by the gradient vector flow in a restricted subimage was exploited. A wide variety of TTC-stained rat brain images captured by a smartphone were generated and employed to evaluate our established frameworks. Experimental results on the segmentation of rat brains and cerebral hemispheres suggested high segmentation accuracy. The developed segmentation algorithms are promising and beneficial in facilitating experimental stroke studies with TTC-stained rat brain images. It is worth investigating the discrimination between the desired brain surfaces and the color tone-oriented segmentation to diminish the gap between human interpretation and machine recognition.

## Figures and Tables

**Table 1:** Rat brain segmentation accuracy analyses based on $\kappa_{D}$ in Figure 5 and Figure 6.

Method	Subject 2	Subject 20	Subject 37
CTM	79.54% 80.47%	76.86% 79.53%	85.22% 77.58%
TPS	82.37% 82.15%	83.22% 81.75%	90.79% 80.02%
Proposed	84.21% 94.95%	94.36% 94.34%	95.24% 93.73%

The preceding number indicates the $\kappa_{D}$ value in Figure 5 and the following number for Figure 6.

**Table 2:** Quantitative performance analyses of the rat brain extraction methods applied to the TTC-stained image. dataset.

Method	$\kappa_{D}\left(\%\right)$	$\kappa_{J}\left(\%\right)$	$\kappa_{c}\left(\%\right)$	$\eta_{st}\left(\%\right)$	$\eta_{sb}\left(\%\right)$
CTM	78.76 ± 9.14	72.55 ± 8.34	45.12 ± 9.95	96.42 ± 2.96	71.32 ± 6.02
TPS	80.87 ± 6.86	73.54 ± 7.02	52.50 ± 8.96	96.13 ± 1.94	73.49 ± 5.02
Proposed	92.33 ± 2.18	85.78 ± 1.26	83.35 ± 2.97	97.73 ± 0.93	85.99 ± 3.12

Listed values are the average ± standard deviation.

**Table 3:** Quantitative performance analyses of the rat hemisphere segmentation results in the TTC-stained image dataset based on similarity measure metrics.

Hemisphere	$\kappa_{D}\left(\%\right)$	$\kappa_{J}\left(\%\right)$	$\kappa_{c}\left(\%\right)$	$\eta_{st}\left(\%\right)$	$\eta_{sb}\;\left(\%\right)$
Left	96.94 ± 0.83	94.07 ± 1.56	93.66 ± 1.78	96.63 ± 1.44	97.26 ± 1.53
Right	97.37 ± 0.76	94.88 ± 1.43	94.58 ± 1.61	96.39 ± 1.55	98.40 ± 1.32
Overall	97.15 ± 0.82	94.47 ± 1.54	94.12 ± 1.75	96.51 ± 1.49	97.83 ± 1.53

Listed values are the average ± standard deviation.

**Table 4:** Quantitative performance evaluation of the rat hemisphere segmentation results in the TTC-stained image dataset based on error analysis metrics.

Hemisphere	${Ν}_{s}$	${Ν}_{g}$	$\Delta_{N}$	$\Delta_{F}$	$\varepsilon_{F\;}\left(\%\right)$
Left	172,566 ± 138,157	173,566 ± 138,805	3574 ± 3567	9781 ± 6949	6.11 ± 1.66
Right	172,570 ± 136,660	175,326 ± 137,243	3853 ± 4136	8668 ± 6508	5.21 ± 1.48
Overall			3714 ± 3831	9224 ± 6698	5.66 ± 1.63

Listed values are the average ± standard deviation.

## Data Availability

The data that support the findings of this study are available for sharing from the corresponding authors upon reasonable request.
